# Synthesizing optimal bias in randomized self-stabilization

**DOI:** 10.1007/s00446-021-00408-4

**Published:** 2021-11-08

**Authors:** Matthias Volk, Borzoo Bonakdarpour, Joost-Pieter Katoen, Saba Aflaki

**Affiliations:** 1 Software Modeling and Verification Group, RWTH Aachen University, Aachen, Germany; 2 Present Address: Formal Methods and Tools, University of Twente, Enschede, The Netherlands; 3 Department of Computer Science, Michigan State University, East Lansing, USA; 4 Index Exchange, Toronto, Canada

**Keywords:** Randomized distributed systems, Self-stabilization, Parameter synthesis, Performance

## Abstract

Randomization is a key concept in distributed computing to tackle impossibility results. This also holds for *self-stabilization* in anonymous networks where coin flips are often used to break symmetry. Although the use of randomization in self-stabilizing algorithms is rather common, it is unclear what the optimal coin bias is so as to minimize the expected convergence time. This paper proposes a technique to automatically synthesize this optimal coin bias. Our algorithm is based on a parameter synthesis approach from the field of probabilistic model checking. It over- and under-approximates a given parameter region and iteratively refines the regions with minimal convergence time up to the desired accuracy. We describe the technique in detail and present a simple parallelization that gives an almost linear speed-up. We show the applicability of our technique to determine the optimal bias for the well-known Herman’s self-stabilizing token ring algorithm. Our synthesis obtains that for small rings, a fair coin is optimal, whereas for larger rings a biased coin is optimal where the bias grows with the ring size. We also analyze a variant of Herman’s algorithm that coincides with the original algorithm but deviates for biased coins. Finally, we show how using *speed reducers* in Herman’s protocol improve the expected convergence time.

## Introduction

*Self-stabilization* [11, 12] is a versatile fault-tolerance technique that ensures the convergence of a distributed system to a good behavior in the presence of arbitrary initializations as well as transient faults. Similar to other techniques in distributed computing, solving certain problems in self-stabilization turn out to be impossible mainly due to the inability of a network to break symmetry. For example, while there exist solutions to the self-stabilizing token circulation problem in networks with a designated process that behaves differently from the others, the problem is impossible to solve in anonymous networks, where all processes are required to execute exactly the same algorithm.

*Randomization* techniques are frequently used in distributed algorithms mainly as a means of breaking symmetry when this is impossible in a deterministic setting. In particular, processes take an action based on a probabilistic distribution and communicate it to the rest of the network. The correctness of such algorithms stems from the zero probability of reaching a state infinitely often where the goal of the algorithm (e.g., leader election, reaching agreement, or self-stabilization) is not met. Even if processes can solve the problem in a deterministic manner (e.g., by employing unique IDs), randomization may lead to breaking symmetry “faster”. Examples of randomized distributed algorithms include seminal algorithms for leader election [23], finding maximal independent set [26], crash consensus [6], and self-stabilization [20]. Most research efforts on randomized distributed algorithms focus on tackling impossibility results and not so much on how fast such algorithms converge to a solution. However, fast convergence is crucial for the performance of the algorithms, as it decreases the number of computation steps and therefore the needed computing resources. In the rest of this section, in order to motivate the problem under investigation in this paper, we first delve deeper in the particular context of impossibility results in self-stabilization and techniques for enhancing performance.

### Coping with impossibility results

**Fig. 1:**
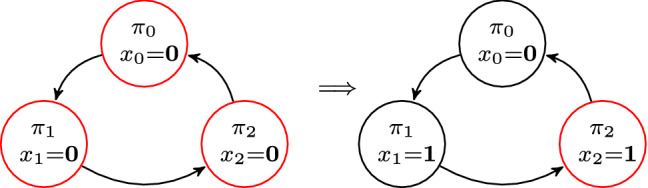
Self-stabilization via Herman’s protocol (color figure online)


Consider Herman’s algorithm [20] as means to tackle the impossibility of self stabilization in anonymous networks in a ring structure with unidirectional communication channels. The protocol for one process is given in Algorithm 1. An example for a ring of three processes is given in Fig. 1. Each process *i* has one bit of memory $x_i$ and can only read the bit of its predecessor $x_{i-1}$. A process *i* has a token if and only if its value is equal to the value of its predecessor, i.e., $x_i = x_{i-1}$. In the left ring of Fig. 1, all three processes have a token (indicated by the red color), because each process has the same value as its predecessor. If a process has a token, it flips a (possibly biased) coin to randomly decide its next value. With probability *p* the bit is set to 0, with probability $1-p$ it is set to 1. If a process does not have a token—i.e., its value is different from its predecessor—it uses the value of its predecessor as its new value. This definition of Herman’s algorithm is also called the *random bit* interpretation [25], because in case of a token the next value is determined by a random bit. The initial configuration of the token ring is determined by a uniform distribution over all possible bit values for all processes. In our example, in the initial configuration each process has a token and, therefore, all processes flip a coin to determine their next value. We assume that process $\pi _0$ sets its bit to 0 and the other processes flip value 1. The resulting configuration is visualized by the ring on the right-hand side of Fig. 1. In the new configuration, only process $\pi _2$ has a token and we have reached a *legitimate configuration*, i.e., exactly one process has a token. Now, the question is whether the value of probability *p* has an impact on the expected recovery time to a legitimate state. Note that we use recovery and convergence interchangeably in the following.
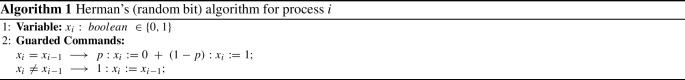

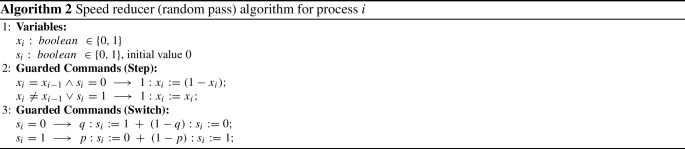

**Fig. 2:**
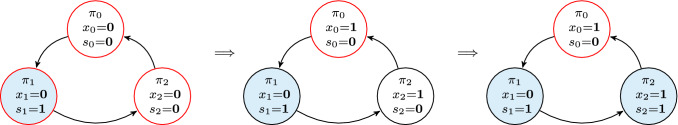
Self-stabilization via speed-reducer protocol (color figure online)

### Enhancing the performance of distributed algorithms

Another application of randomization is in increasing the performance of distributed algorithms. More specifically, one can compose the (possibly randomized) distributed algorithm with a randomized algorithm, called a *speed reducer* [13], that controls the spread of a fault by reducing the speed of, e.g., multiple tokens traveling in a ring. In particular, one can solve the self-stabilization problem by (randomly) giving some processes a different behavior. Processes in “normal” mode always pass along their token. Processes in “speed reducer” mode always keep their token and do not forward it. This protocol for one process is described in Algorithm 2, where Step and Switch phase are executed sequentially. An example for a ring of three processes is given in Fig. 2. Each process has an additional bit $s_{i}$ to store the current mode. The process is in speed-reducer mode, if $s_{i}=1$. Each process starts in normal mode. In our example configuration, all processes again hold a token and process $\pi _1$ is in speed-reducer mode (as indicated by the blue shading). If a process holds a token and is not in speed-reducer mode, it forwards the token by flipping its bit. If a process does not hold a token or is in speed-reducer mode, it does nothing—and possibly keeps the token. In our example, processes $\pi _0$ and $\pi _2$ hold a token and are in normal mode. They thus flip their bit. Process $\pi _1$ is in speed-reducer mode and keeps its value. In the resulting configuration (depicted in the middle of Fig. 2), only $\pi _0$ has one token. Intuitively, during the computational step $\pi _1$ held two tokens—one forwarded token from $\pi _0$ and its own token. Whenever a process has two tokens, both vanish. This results in $\pi _1$ having no token. After all processes have performed a step—i.e., either flipping their bit or doing nothing—each process flips a coin to decide whether its mode should be switched. Processes switch from normal to speed-reducer mode with probability *q*, and switch in the reverse direction with probability *p*. In our example, processes $\pi _0$ and $\pi _1$ stay in their current mode and $\pi _2$ switches to speed-reducer mode. It is of interest now to know which bias of *p* *and* *q* results in the best expected performance of the speed reducer.

### Our contribution—synthesizing optimal bias

A crucial contributing factor in the performance of randomized distributed algorithms is the choice of the employed probability distribution. For instance, an empirical study [25] shows a counter-intuitive result: using a biased coin in Herman’s algorithm [20] for larger rings leads to faster average recovery time than using a fair coin. We begin with the premise that for most randomized distributed algorithms, it is unclear what choice of a probability distribution results in faster termination of the algorithm, or higher probability of obtaining a correct solution. Lack of this knowledge is primarily due to the subtlety of analytical methods (e.g., closed-form formulas that provide optimal probabilities by using algebraic techniques) to characterize the performance of randomized distributed algorithms. The result in [25] motivates an even deeper and more challenging research problem that we call *synthesizing optimal bias*:

*Given a randomized distributed algorithm, is it possible to automatically synthesize a probability distribution resulting in the best performance for the algorithm without changing its control flow?*

This paper addresses the aforementioned problem in the context of randomized self-stabilizing systems. Following the results in [14, 15], our choice of performance metric is *average recovery time*. In particular, we propose a fully automated technique that takes as input a randomized self-stabilizing algorithm, where processes execute their actions with some probability, and generates as output the probability value based on which, the self-stabilizing algorithm exhibits the *minimum* average recovery time. Our technique:

- First transforms a randomized self-stabilizing algorithm into a parametric Markov chain (PMC). A PMC is represented by a symbolic transition probability matrix (TPM), where each entry is a function over the parameters describing the probability of one-step reachability of each state from other states.
- Next, to compute the optimal bias, we compute over- and under-approximations of the average recovery time for all parameter values inside a given parameter region $\mathcal {R} \subseteq [0, 1]$. By iteratively refining the regions which lead to small convergence times, the optimal probability values can be approximated up to the desired precision. To this end, we extend the existing techniques of approximation via *parameter lifting* [9, 29]. Our algorithm also effectively parallelizes the computation of independent iterations, resulting in faster computation of the optimal bias using multi-core platforms.

We emphasize that our technique does not change the semantics (i.e., the structure of the transition system) of the randomized self-stabilizing protocol. We merely identify the probabilities that result in the best average recovery time. Hence, our technique can potentially be applied in dealing with network parameters and more importantly in identifying the best distribution for probabilistic schedulers. We also note that our techniques are completely independent of the probabilistic scheduling policy in the underlying PMC. In case the type of scheduler is of importance, one can combine the techniques introduced in this paper with the composition method in [1].

Our synthesis algorithm is fully implemented and we demonstrate the effectiveness of our approach using two detailed case studies: (1) Herman’s randomized self-stabilizing token ring algorithm [20], and (2) the role of speed reducers [13] to improve convergence time. While we give an in-depth analysis of the performance of these algorithms, the summary of our result is as follows. For Herman’s algorithm, for different network sizes, we synthesize the probabilities that result in nearly minimum average convergence time. In particular, we show that while for smaller networks a fair coin results in the best average convergence time, for larger networks a biased coin is advantageous. This confirms the findings in [25] by *synthesizing* the optimal bias in an automated manner, rather than by sweeping over the analysis results for multiple variants (one per bias). In the case of speed reducers, our experiments show that composing a speed reducer with two variants of Herman’s algorithm (i.e., random bit and random pass) improves the performance of both. Experiments with Dijkstra’s deterministic *k*-state token ring algorithm [11] reveal that speed reducers do not yield better average recovery time (for rings of size 3 and 5).

*Comparison to the conference version* This work is based upon [2]. While [2] presented three different solution approaches to the parameter synthesis problem, in this work we focus on the most promising approach based on parameter lifting and significantly improve it compared to the original approach. Moreover, we considerably extend the evaluation performed in [2] by creating and analyzing new self-stabilizing algorithms based on speed-reducers. In summary, we make the following new contributions in this work:

- Major improvements of the approximation approach by exploiting parallelization, using symbolic model building and performing sampling.
- Parameter synthesis for optimal bias in Herman’s algorithm and several extensions based on speed reducers.
- In-depth evaluation of the implemented approximation approach and the considered self-stabilizing protocols.

*Organization* The rest of the paper is organized as follows. Section 2 describes our computational model and introduces (parametric) Markov chains. Section 3 presents the concept of self-stabilization and formally states our fine-tuning problem. Section 4 introduces the approximation approach using the *parameter lifting algorithm* [29] and provides details on the improved implementation. Section 5 presents a detailed evaluation of the approximation approach and parameter synthesis for several self-stabilizing algorithms. Related work is discussed in Sect. 6. Finally, we make concluding remarks and discuss future work in Sect. 7.

## Computational model

This section introduces the computational model of distributed algorithms.

### Distributed programs

A *distributed program* ${\mathcal {D}}{\mathcal {P}}$ consists of a finite set $\varPi$ of *processes* and a finite set $V$ of discrete *variables*. The *state space* $S_{{\mathcal {D}}{\mathcal {P}}}$ of ${\mathcal {D}}{\mathcal {P}}$ is given by:

$$\begin{aligned} S_{{\mathcal {D}}{\mathcal {P}}} = \prod _{v \in V} D_{v}, \end{aligned}$$

where $D_{v}$ is the finite domain of variable *v*. A *state* $s \in S_{{\mathcal {D}}{\mathcal {P}}}$ is denoted as vector $s=\langle v_1,\dots ,v_{\vert {V}\vert } \rangle .$ A *state predicate* is a subset of $S_{{\mathcal {D}}{\mathcal {P}}}$.

Each process $\pi \in \varPi$ is associated with a *read-set* $R_{\pi } \subseteq V$ of variables read by $\pi$ and a *write-set* $W_{\pi } \subseteq V$. Following the *shared-memory* model, we require $W_{\pi } \subseteq R_{\pi }$ and $W_{\pi } \cap W_{\pi '} = \emptyset$ for all $\pi , \pi ' \in \varPi$ with $\pi \ne \pi '$. Processes $\pi \ne \pi '$ are called *neighbors* if $R_{\pi } \cap R_{\pi '} \ne \emptyset$.

The behavior of process $\pi$ is described by a finite set $\mathcal {G}_\pi$ of *probabilistic guarded commands* of the form:

$$\begin{aligned} \langle label \rangle \; :\; \langle guard \rangle \; \rightarrow \;&p_1:\langle statement_1\rangle \; +\; \cdots \; +\\&p_n:\langle statement_n\rangle ; \end{aligned}$$

where *guard* is a Boolean expression over $R_{\pi }$ and

$$\begin{aligned} \sum _{i=1}^n p_i = 1 \end{aligned}$$

where $p_i$ can be either a concrete value or a parameter. If *guard* is true, $statement_i$ is executed with probability $p_i$, updating the variables in $W_{\pi }$ while moving from state $s \in S_{{\mathcal {D}}{\mathcal {P}}}$ to $s' \in S_{{\mathcal {D}}{\mathcal {P}}}$. Thus, when a guard *g* is enabled at state *s*, a set *g*(*s*) of *successor states* can be reached, generated by executing all $statement_i$. If multiple guards hold, an enabled one is chosen with uniform probability.

#### Example 1

**Fig. 3:**
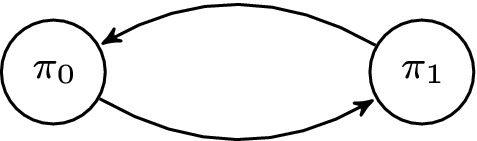
Example of a ring of two processes

Consider a simple token-passing algorithm described by the guarded commands in Algorithm 1 for process $\pi _i$, $i \in \{0,1\}$, see Fig. 3. For the sake of simplicity, we assume a process $\pi _i$ to have a token if and only if $x_i = 1$. Each process $\pi _i$ has a variable $x_i$ with $D_{x_i}=\{0,1\}$. In state $\langle 0, 1 \rangle$, variable $x_1$ has value 1, i.e., process $\pi _1$ has a token. Both processes simultaneously execute the guarded command (i.e., a synchronous timing model):

$$\begin{aligned} g:\;\; x_i\ne x_{i-1}\; \longrightarrow \; 1: x_i:=x_{i-1}; \end{aligned}$$

and flip their value resulting in state $g(\langle 0, 1 \rangle ) = \{ \langle 1, 0 \rangle \}$.

States without outgoing transitions are assumed to be equipped with a self-loop.

#### Definition 1

(*Computation*) A *computation* ${\sigma }$ of a distributed program ${\mathcal {D}}{\mathcal {P}}$ is an infinite sequence of states:

$$\begin{aligned} {\sigma }= s_{0} \, s_{1} \, s_{2} \cdots \in S_{{\mathcal {D}}{\mathcal {P}}}^\omega , \end{aligned}$$

where for all $i \ge 0$, $s_{i+1} \in g(s_i)$ for some command *g*.

### Markov chains

The operational behaviour of distributed programs is described by *Markov chains* [4], i.e., finite automata whose transitions are equipped with probabilities. Let ${ Dist}(S)$ denote the set of discrete probability distributions over the set *S*.

#### Definition 2

(*DTMC*) A *Discrete-time Markov Chain* is a tuple $\mathcal {D}=(S,{\iota _{init}},\mathbf {P})$ where

- *S* is a finite set of *states*.
- ${\iota _{init}}\in { Dist}(S)$ is the *initial state distribution*.
- $\mathbf {P}: S \rightarrow { Dist}(S)$ is the *transition probability matrix (TPM).*

A state $s \in S$ with ${\iota _{init}}(s) > 0$ is *initial*. Note that in self-stabilizing systems, any state can be initial. Our parameter synthesis algorithm uses an extension of DTMCs where in each state there is a choice of possible distribution. This model is known as MDPs [4, 28].

#### Definition 3

(*MDP*) A *Markov Decision Process* is a tuple $(S,{\iota _{init}},{ Steps})$ with *S* and ${\iota _{init}}$ as above, and ${ Steps}: S \rightarrow 2^{\small { Dist}(S)}$ assigns to each state *s* a finite, non-empty set ${ Steps}(s)$ of distributions on *S*.

An MDP with $| { Steps}(s) | = 1$ for any state *s* is a DTMC.

A *parametric Markov chain (PMC)* [5, 8] is a DTMC where the transition probabilities are multi-variate polynomials over a fixed set of parameters. Let $U=\{p_1,\ldots ,$ $p_k\}$ be a finite set of parameters and $F_{U}$ denote the set of multi-variate polynomials over *U*.

#### Definition 4

(*PMC*) A *parametric Markov chain* over *U* is a tuple $\mathcal {P}=(S,U,{\iota _{init}},\mathbf {P})$ with *S* as before, and

- ${\iota _{init}}: S \rightarrow F_{U}$ is the initial state distribution.
- $\mathbf {P}: S \times S \rightarrow F_{U}$ is the transition probability matrix.

An *evaluation function* $eval: U \rightarrow \mathbb {Q}$ assigns rational values to parameters in *U*. For polynomial $f \in F_{U}$, $eval(f)$ denotes the value obtained by replacing each parameter $p_i$ in *f* by $eval(p_i)$.

#### Definition 5

(*Instantiated PMC*) The *instantiated PMC* for PMC $\mathcal {P}=(S,U,{\iota _{init}},\mathbf {P})$ under evaluation function $eval$ is  where

for all $s \in S$ and
- $$\mathbf {P}_{eval}(s,s') = eval(\mathbf {P}(s,s'))$$ for all $$s, s' \in S$$.

The evaluation function $eval$ is *valid* for $\mathcal {P}$ if the instantiated PMC $\mathcal {P}[eval]$ is a DTMC, i.e., if:The transition system of a distributed program can be straightforwardly modeled by a PMC. The TPM of a process can be trivially derived from its set of probabilistic guarded commands. We refrain from providing the technical details of this mapping, and instead provide an example. The reader may consult^1^ for a detailed semantics of probabilistic guarded commands.

#### Example 2

**Fig. 4:**
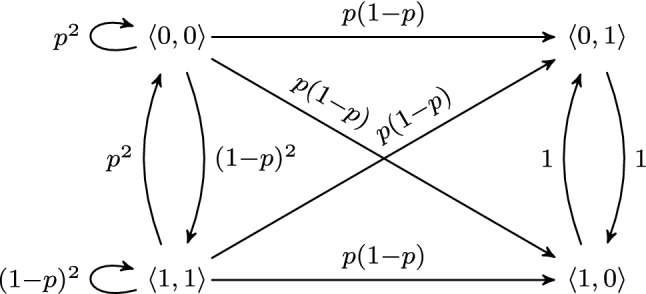
PMC for simple token-passing algorithm

The TPM of the algorithm for the two processes in Example 1 is as follows:A visualization of the PMC is given in Fig. 4. For example, in state $\langle 0, 0 \rangle$ both processes have the same value as their preceding process. Therefore, each process flips a coin and changes its value accordingly. One outcome is that both processes change their value to 1 with probability $1-p$. Thus, there is a transition from state $\langle 0, 0 \rangle$ to state $\langle 1, 1 \rangle$ with probability $(1-p)^2$.

## Problem statement

In this section, we formally state the problem of synthesis of optimal bias. We start with introducing the notion of probabilistic self-stabilization.

### Self-stabilization and recovery time

In this section, we only consider distributed programs with concrete probabilities—and no parameters.

#### Definition 6

(*Self-stabilization* [12]) Let $LS$ be a state predicate denoting the set of *legitimate states*. A distributed program ${\mathcal {D}}{\mathcal {P}}$ is called *self-stabilizing* for $LS$ iff it satisfies the following two conditions:

1. *Strong convergence*: starting from any arbitrary initial state, every computation converges to a legitimate state in a finite number of steps.
2. *Closure*: after reaching a legitimate state, the computation is guaranteed to remain in $LS$ as long as no fault occurs.

Strong convergence is a rather strict condition and has led to several impossibility results [3]. Therefore, *probabilistic convergence* and hence, *probabilistic self-stabilization* under a probabilistic scheduler^2^ was introduced [20]. A distributed program ${\mathcal {D}}{\mathcal {P}}$ *probabilistically converges* if starting from any arbitrary state it can reach a legitimate state *with probability one*.

#### Example 3

In our running example cf. Fig. 4, $LS$ consists of all states in which exactly one process holds a token, i.e., $x_i = 1$ for one and only one *i*:

$$\begin{aligned} LS =\{s_1=\langle 0,1 \rangle , s_2=\langle 1,0 \rangle \}. \end{aligned}$$

This paper focuses on probabilistic self-stabilization. We represent a probabilistic self-stabilizing program ${\mathcal {D}}{\mathcal {P}}$ with legitimate states $LS$ by a DTMC $\mathcal {D}_{{\mathcal {D}}{\mathcal {P}}} = (S,{\iota _{init}},\mathbf {P})$, where $LS \subseteq S$ and ${\iota _{init}}(s) = \frac{1}{|S|}$, i.e., any arbitrary state is an initial state with equal probability.

#### Definition 7

(*Recovery path*) Let ${\mathcal {D}}{\mathcal {P}}$ be a probabilistic self-stabilizing program, $\mathcal {D}_{{\mathcal {D}}{\mathcal {P}}} = (S,{\iota _{init}},\mathbf {P})$ be the corresponding DTMC. A *recovery path* of $\mathcal {D}_{{\mathcal {D}}{\mathcal {P}}}$ from state $s \in S$ is a finite computation ${\sigma }_r= s_0 s_1 \cdots s_n$ with

- $$s_0 = s$$,
- $\mathbf {P}(s_i, s_{i+1}) > 0$ and $s_i \not \in LS$ for all $0\le i <n$, and
- $$s_n \in LS $$.

Let ${\sigma }_r(s)$ denote the set of all recovery paths from state *s*. The *recovery probability* from a state *s* is

$$\begin{aligned} \mathbb {P}(s) = \sum _{{\sigma }\in {\sigma }_r(s)} \mathbb {P}({\sigma }) \quad \text{ with } \quad \mathbb {P}({\sigma }) = \prod _{0 \le i < n} \mathbf {P}(s_i, s_{i+1}). \end{aligned}$$

We now have everything in place to formally define probabilistic self-stabilization.

#### Definition 8

(*Probabilistic self-stabilization*) Let $LS$ be the *legitimate states* and ${\mathcal {D}}{\mathcal {P}}$ a distributed program. ${\mathcal {D}}{\mathcal {P}}$ is *probabilistic self-stabilizing* iff:

1. *Probabilistic recovery*: $\mathbb {P}(s) = 1$ for all $s \in S$, and
2. *Closure*: for all $s \in LS$, all processes $\pi \in \varPi$ and all guarded commands $g \in \mathcal {G}_\pi$, we have $g(s) \subseteq LS$.

The *recovery time* of a recovery path ${\sigma }_r= s_0 \cdots s_n$ is $\mathbf {R}({\sigma }_r) = n$. The *expected recovery time for a state* *s* is

$$\begin{aligned} \mathbf {R}(s) = \sum _{{\sigma }\in {\sigma }_r(s)} \mathbb {P}({\sigma }) \cdot \mathbf {R}({\sigma }) \end{aligned}$$

and the *expected recovery time for a program* ${\mathcal {D}}{\mathcal {P}}$ with corresponding DTMC $\mathcal {D}_{{\mathcal {D}}{\mathcal {P}}} = (S,{\iota _{init}},\mathbf {P})$ is

$$\begin{aligned} ERT (\mathcal {D}_{{\mathcal {D}}{\mathcal {P}}})=\sum _{s \in S} {\iota _{init}}(s) \cdot \mathbf {R}(s). \end{aligned}$$

### The optimal bias parameter synthesis problem

The synthesis problem takes as input a distributed program ${\mathcal {D}}{\mathcal {P}}$ (with parametric probabilities) which is modeled by a PMC $\mathcal {P}_{{\mathcal {D}}{\mathcal {P}}}$. We consider all evaluation functions $eval$ which are valid for $\mathcal {P}_{{\mathcal {D}}{\mathcal {P}}}$. Each instantiated PMC $\mathcal {P}_{{\mathcal {D}}{\mathcal {P}}}[eval]$ and corresponding distributed program (with concrete probabilities) satisfy Definition 8. The goal is to find the evaluation function $eval$ for which the expected recovery time of $\mathcal {P}_{{\mathcal {D}}{\mathcal {P}}}[eval]$ is minimal. 
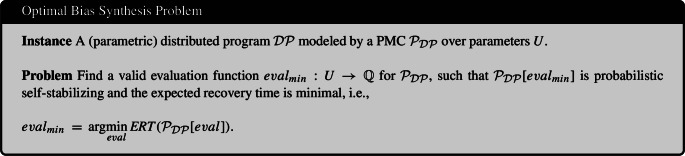


An approximate version of this problem requires to find the minimal expected recovery time up to a given inaccuracy $\epsilon$. This problem variant is useful to consider for practical efficiency purposes. Let $ERT _{min} = ERT (\mathcal {P}_{{\mathcal {D}}{\mathcal {P}}}[eval_{min}])$ be the minimal expected recovery time for the optimal evaluation function $eval_{min}$. The goal of the *approximate* optimal bias synthesis problem is to find an evaluation function $eval_{approx}$ which is valid for $\mathcal {P}_{{\mathcal {D}}{\mathcal {P}}}$, the instantiated PMC $\mathcal {P}_{{\mathcal {D}}{\mathcal {P}}}[eval_{approx}]$ is probabilistic self-stabilizing and the difference to the minimal expected recovery time is at most $\epsilon$, i.e.,

$$\begin{aligned} ERT (\mathcal {P}_{{\mathcal {D}}{\mathcal {P}}}[eval_{approx}]) - ERT _{min} \le \epsilon . \end{aligned}$$

## Approximation via parameter lifting

The optimal bias synthesis problem can be seen as a parameter synthesis problem on parametric Markov chains [8, 24]. In previous work [2], we presented three different approaches to synthesize optimal parameter values:

1. using symbolic linear algebraic methods,
2. computing rational functions via state elimination,
3. approximation via parameter lifting.

The first two approaches compute exact solutions and are very similar. Both approaches compute a closed form of the expected recovery time in terms of a rational function over the parameters. Finding the minima of this rational function yields the desired exact optimal parameter values. However, as shown in [2], both approaches do not scale well for larger model sizes, because the rational functions can grow exponential in the number of parameters and polynomial in the number of states [22]. The third approach approximates the optimal values which scales better. We therefore focus on this approximation approach in the following.

### Parameter lifting

The approximation approach is based on the *parameter lifting algorithm (PLA)* first described in [29]. The idea is to lift the parameter synthesis problem of a parametric Markov chain (PMC) to a model-checking problem of a Markov decision process (MDP).

Assume that we have a PMC with *n* parameters $p_1, \dots , p_n$ with possible values $p_i \in [l_i, u_i] \subseteq \mathbb {Q}$. The Cartesian product of the intervals of all parameters $[l_1, u_1] \times \dots \times [l_n, u_n]$ is called a *region*. For example $[0.2, 0.8] \times [0.3, 0.5]$ is a region for two parameters. In the general case, a region is an *n*-dimensional hyper-rectangle.

**Fig. 5:**
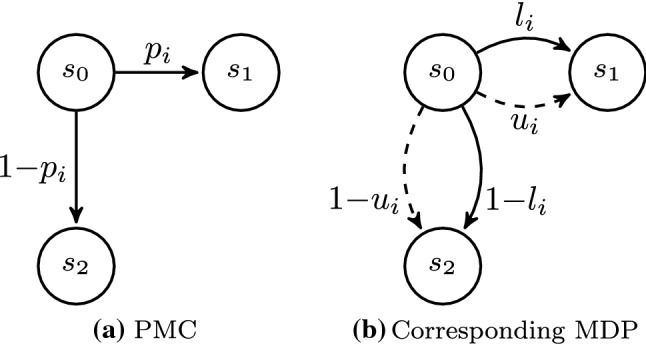
PLA translation

The key observation in PMCs is that for transition probabilities of the form $p_i$ or $1-p_i$, i.e., linear functions, the minimal/maximal probabilities to reach a set of target states occur at the extremal values of the parameters. To obtain the minimal/maximal reachability probability (or expected recovery time) it, therefore, suffices to consider the minimal/maximal values ($l_i$ or $u_i$) of the parameter $p_i$ instead of all (infinitely many) values. Thus, we translate the PMC into an MDP by replacing each parametric transition $p_i$ with the two actions $l_i$ and $u_i$. This translation is illustrated in Fig. 5, where the PMC is depicted in Fig. 5a and the resulting MDP in Fig. 5b. The MDP contains two different actions indicated by the straight and dashed transitions. The first action (straight transitions) uses the minimal bound $l_i$ of the parameter $p_i$, the second action (dashed transitions) uses the maximal bound $u_i$.

By replacing the parameters with their bounds, the dependencies between states which have the same parameter on outgoing transitions are lost. In the PMC on all these states the parameter must have the same value. However, in the MDP we can choose to use the lower bound for one state and use the upper bound on a different state. Thus, PLA does not obtain precise results, but instead over- and under-approximations of the exact result. Model checking on the MDP yields the maximal and minimal probabilities for a given region. The approximation will become more precise if the lower and upper bound of the parameters become close, i.e., the region becomes smaller. Thus, if the obtained result is not precise enough yet, we use smaller regions by *splitting* a region. For example, instead of analyzing the region [0.3, 0.5], we can analyze the regions [0.3, 0.4] and [0.4, 0.5] instead.

**Fig. 6:**
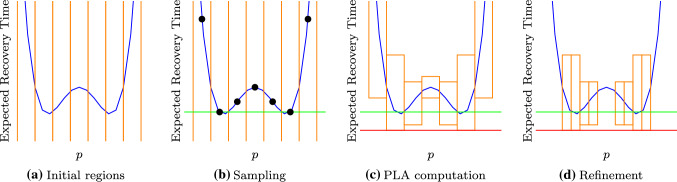
Steps of the approximate optimal bias synthesis algorithm (color figure online)

### Algorithm

We use the *parameter lifting algorithm (PLA)* to compute regions containing the optimal parameter values for our approximate optimal bias synthesis problem for distributed algorithms. By iteratively refining and tightening these regions we can approximate the optimal values up to a desired precision. To ease the presentation, we only consider one parameter in the following, but the algorithm is applicable to several parameters in the same manner.

The general steps of the approximate optimal bias synthesis algorithm are visualized in Fig. 6. The exact probability function is depicted in blue. Our goal is to approximate the minima of this function. In our application setting, this corresponds to minimize the ERT. We start with a PMC, a precision bound $\epsilon$ and an initial parameter region $\mathcal {R}_0 \subseteq (0, 1)$. The initial region excludes the bounds 0 and 1 as for those parameter values transitions with probabilities of the form *p* or $1-p$ vanish and the graph of the PMC is not preserved. We note that the PLA approach is only sound if the extremal values for all transition probabilities occur at the region bounds. While this is usually ensured by only allowing linear transition probabilities, we support arbitrary polynomials here. For each polynomial occurring as a transition probability, we compute the parameter values which yield minimal/maximal values. We split the initial region at these values into multiple regions and therefore ensure that all extremal values can only occur at the region bounds. In Fig. 6a, we indicate the initial regions by the orange lines.

For each region, we compute a sample point by instantiating the PMC with the corresponding parameter values. The sample points for each region are indicated by black dots in Fig. 6b. Instantiating the PMC yields a DTMC which can be analyzed by model-checking techniques to obtain the exact ERT for a concrete parameter value. We use the minimal ERT over all sample points as the new upper bound on the minimal ERT. This upper bound is indicated by the green line and we know that the optimal ERT must lie on or below this line.

In the next step, we translate the PMC into an MDP and compute the over- and under-approximation for each region via PLA. The resulting upper and lower bounds for each region are given by the orange boxes in Fig. 6c. We use the minimal lower bound over all regions as the new lower bound on the minimal ERT. This lower bound is indicated by the red line and we know that the optimal ERT must lie in between the green and red line.

Each region with a lower bound greater than the (green) upper bound on the ERT can be discarded, because even in the best case the sampled ERT can never be reached. In our example, the two outer regions and the middle region can all be removed. For the remaining four regions, we need more precise PLA approximations to narrow down the optimal parameter values. We, therefore, split each region in half and end up with 8 smaller regions as given in Fig. 6d. These three steps—sampling, PLA, and refinement—are iterated. Our approach terminates, if the difference between the upper (green) and lower (red) bound of the ERT is smaller than the desired precision $\epsilon$. Then, the parameter evaluation corresponding to the upper bound is a solution for the approximate synthesis problem.

### Parallelization

A natural way to improve the running times of the synthesis algorithm is to exploit the availability of multiple CPU cores on modern computer systems. Our algorithm can profit from parallelization in the sampling and PLA steps. For each CPU core, we create a new instance of the model checker with the given PMC. These model checkers are used in a *thread pool* configuration. That means each new task—sampling or PLA—is appended to a work queue of the thread pool. Each idle process takes the next task from the work queue and performs the required operation. For both sampling and PLA, we create tasks for each region and let the thread pool handle them in parallel. The only communication between the main process and the worker processes is the considered sample point or region as input and the resulting ERT as output. Thus, both sampling and PLA can easily be parallelized with almost no overhead.

The *parallelized* synthesis algorithm is presented in detail in Algorithm 3. The algorithm gets as input a distributed program ${\mathcal {D}}{\mathcal {P}}$ of a self-stabilizing algorithm, an initial region $\mathcal {R}_{0}$ and the desired precision $\epsilon$. It returns the regions containing the optimal parameter values, the approximation bounds for the optimal ERT and the parameter values which yield the upper bound.
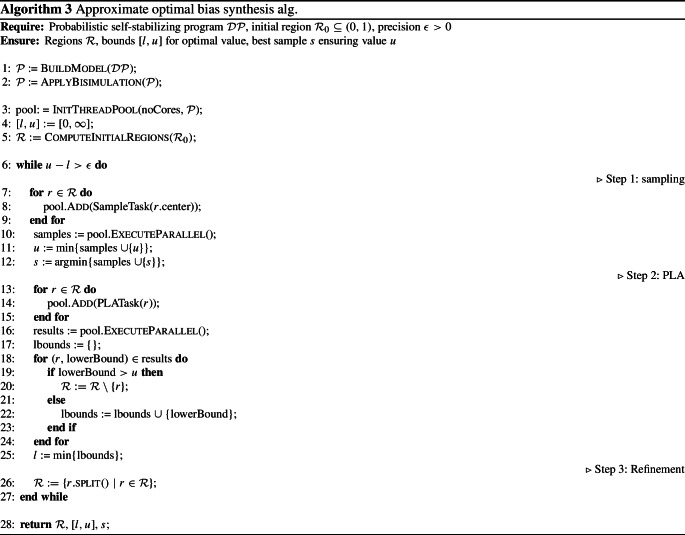


We first build the PMC $\mathcal {P}$ from the distributed program ${\mathcal {D}}{\mathcal {P}}$ (line 1) and apply bisimulation minimization [18] to obtain a smaller PMC (line 2). Next, we initialize the thread pool and the bounds for the ERT (lines 3–4). The list of initial regions is computed by taking the extremal values of the occurring polynomials into account (line 5). We iterate the three steps—sampling, PLA and refinement—until the desired precision of the ERT approximation is reached (line 6). In the sampling step, we generate a task for each center point of a region (line 8) and execute these tasks in parallel on all available cores (line 10). The minimum over all sampled ERTs and the previous upper bound is the new upper bound for the ERT (line 11) and *s* stores the corresponding sample point (line 12). For the PLA step, we again create tasks for each region (line 14) and execute them in parallel (line 16). If the resulting lower bound of a region is larger than the current upper bound of the ERT, we discard this region as it cannot contain the optimal value (line 20). Otherwise, we add the lower bound to the list of lower bounds (line 22). After considering all region results, we set the current lower bound of the ERT to the minimum of the lower bounds among all regions (line 25). In the third step, we refine the remaining regions by splitting each region and creating new smaller regions (line 26). If the desired precision $\epsilon$ of the approximation [*l*, *u*] is reached, the loop terminates and we return the optimal regions $\mathcal {R}$, the bounds of the ERT and the parameter values *s* ensuring the upper bound (line 28).

#### Theorem 1

Algorithm 3 terminates and solves the approximate optimal bias synthesis problem.

#### Proof

(Sketch) We start with the correctness. The iteration in Algorithm 3 stops, if $$u-l \le \epsilon $$ (cf. line 6). That means the difference between the upper bound *u* obtained from sample point *s* (lines 11–12) and the lower bound *l* is at most $$\epsilon $$. As *l* is an under-approximation computed by PLA (line 25), the minimum $$ ERT _{min}$$ must lie within [*l*, *u*] and in particular, $$u - ERT _{min} \le \epsilon $$. Thus, the evaluation corresponding to sample point *s* is a solution of the approximate synthesis problem.

We show that Algorithm 3 terminates. In each iteration, all remaining regions are split into smaller regions (line 26). As the regions become smaller, the under-approximations computed by PLA become more precise for each region, i.e., closer to the exact minimum. The sample point of each region also yields a value closer to the minimum, because the distance between this center point and the region bounds decreases in each iteration. In other words, the regions converge to point intervals and therefore the upper and lower bounds converge to the same value. Overall, the difference between the sampled value (upper bound) and the value obtained by PLA (lower bound) becomes smaller when the regions become smaller. Through splitting, the difference between the overall upper bound *u* and the lower bound *l* decreases and thus, the algorithm terminates. $\square$

Note that the upper bounds on regions as computed by PLA are never used in our synthesis algorithm. In contrast to the previous algorithm of [2], upper bounds via PLA are not computed. Instead, sampling is used to obtain upper bounds which is faster to compute and gives better results.

Using upper bounds however allows to ensure that all remaining regions yield ERT values within $\epsilon$ of the optimal ERT. With samples, we can only guarantee that the best sample point yields an ERT which is at most $\epsilon$ away from the optimal ERT. For the remaining regions no such claim is possible and we only know that the regions contain the optimal parameter values.

## Evaluation

### Experimental setup

In the following, we evaluate our parameter synthesis algorithm in detail. Our implementation and the model files for the considered self-stabilizing algorithms are publicly available online.^3^

All computations were performed on an Intel Xeon Platinum 8160 with up to 24 cores, each running on 2.1 GHz and up to 768 GB of memory. A timeout of 12 h was used unless indicated otherwise.

The approximate optimal bias synthesis algorithm is implemented as a Python script and was executed using CPython version 3.6.8. The script uses the probabilistic model checker Storm [10] (in version 1.4.1) as a back end for model building, sampling and PLA calls. We use the Python bindings of Storm called Stormpy^4^ (in version 1.4.1). Stormpy offered us a great flexibility when developing the algorithm while simultaneously allowing us to use the state-of-the-art model-checking algorithms without performance loss.

### Herman’s algorithm with biased coins

We first study Herman’s token circulation algorithm [20] (cf. Algorithm 1). Kwiatkowska et al. [25] showed by repeated model checking of various instantiations of the coin bias that using a biased coin in Herman’s algorithm improves the *worst-case* performance of self-stabilization. Here, we *synthesize* the optimal coin bias for minimal *expected recovery time* in a fully automatic approach.

**Fig. 7:**
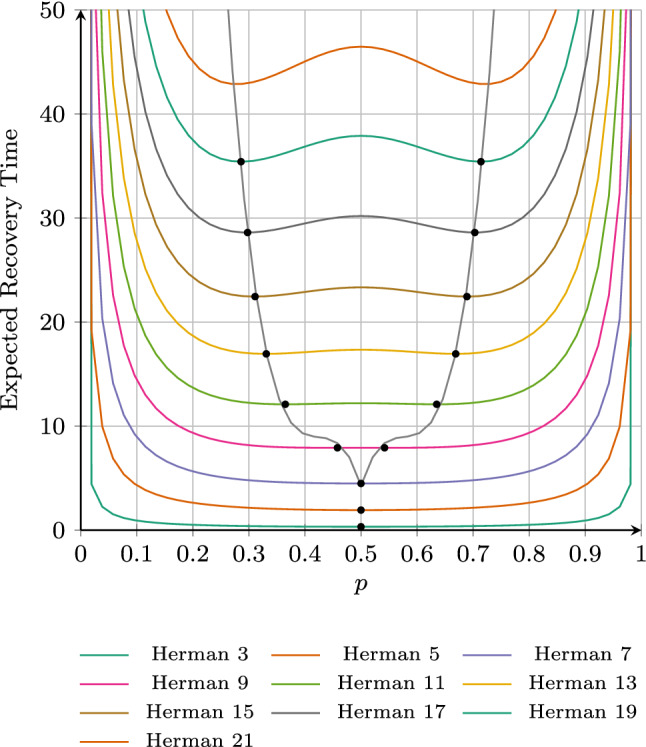
Expected recovery times for Herman’s protocol

Figure 7 depicts the relationship between the coin probability *p* and the expected recovery time (ERT) for different number of processes in the ring. We indicate the optimal parameter values of *p* (as computed by our algorithm) by black dots. Note that our algorithm returns parameter regions, but for presentation purpose we depict a sample point from within the region instead.

**Table 1:** Results for automated synthesis of $\text {ERT}_\text {opt}$ and $p_\text {opt}$ in Herman’s protocol

Model	PMC		PMC (bisimulation)		Results for Symb. P4 (1E−2)		
Size	# States	# Trans.	# States	# Trans.	${\mathrm{ERT}_\mathrm{opt}}/{{{WC}_{opt}}}$	${\mathrm{p}_\mathrm{opt}}$	Time
3	8	28	2	3	[0.333, 0.333]	[0.000, 1.000]	1 s
					*[1.333, 1.333]*	*[0.000, 1.000]*	*1 s*
5	32	244	4	11	[1.933, 1.933]	[0.211, 0.789]	4 s
					*[3.200, 3.200]*	*[0.211, 0.789]*	*2 s*
7	128	2188	15	122	[4.485, 4.493]	[0.455, 0.545]	1 s
					*[6.848, 6.857]*	*[0.469, 0.531]*	*2 s*
9	512	19,684	54	1149	[7.914, 7.921]	[0.419, 0.581]	2 s
					*[11.994, 12.000]*	*[0.498, 0.502]*	*3 s*
11	2048	177,148	181	9900	[12.097, 12.102]	[0.352, 0.382], [0.618, 0.648]	5 s
					*[16.586, 16.595]*	*[0.280, 0.297], [0.703, 0.720]*	*8 s*
13	8192	1,594,324	624	84,669	[16.942, 16.949]	[0.322, 0.344], [0.656, 0.678]	15 s
					*[22.492, 22.499]*	*[0.259, 0.273], [0.727, 0.741]*	*16 s*
15	32,768	14,348,908	2182	713,042	[22.445, 22.453]	[0.301, 0.319], [0.681, 0.699]	105 s
					*[30.422, 30.430]*	*[0.256, 0.267], [0.733, 0.744]*	*80 s*
17	131,072	129,140,164	7702	6,046,299	[28.603, 28.610]	[0.291, 0.304], [0.696, 0.709]	1009 s
					*[37.318, 37.323]*	*[0.248, 0.256], [0.744, 0.753]*	*728 s*
19	524,288	1,162,261,468	27,585	51,072,534	[35.406, 35.416]	[0.279, 0.292], [0.708, 0.721]	8215 s
					*[45.887, 45.894]*	*[0.242, 0.251], [0.749, 0.758]*	*7690 s*
21	2,097,152	10,460,353,204	99,868	431,475,455	–	–	> 12 h
					–	–	> *12 h*

As can be seen, for up to 7 processes, a fair coin with $p=0.5$ yields optimal results. However, for 9 processes and beyond, using a biased coin is optimal. Because of the symmetric nature of the protocol for every configuration there are two symmetrical optimal solutions. On increasing the number of processes, the optimal values for *p* move further away from the fair coin with $p=0.5$. Thus, an interesting question is what the optimal values are for larger number of processes. To this purpose, we fitted a function to the given optimal values and obtained best results when using a cubic function for each “side” of the optimal values. The corresponding two functions are depicted in black in the plot and nearly perfectly fit the optimal parameter values. Looking at the cubic function, we conjecture that the parameter values will converge to the extremal values 0 and 1 in the limit. However, to make an informed guess for the convergence and a better estimation of the quality of our fitted function, some more results are necessary. Obtaining results for larger number of processes, however, requires immense computational efforts. For 21 processes we were not able to approximate optimal values due to memory exhaustion (with 768 GB available) and, therefore, no black dot is shown.

A detailed overview of the results for Herman’s protocol is given in Table 1. The first column depicts the number of processes in the network. The next two columns give the number of states and transitions of the resulting PMC. The fourth and fifth columns give the same measures but after applying bisimulation minimization on the PMC. The results of the parameter synthesis algorithm (Algorithm 3 applied to the minimized PMC) are given in columns six to eight. The algorithm was executed in parallel with four processes and used a symbolic PMC for the initial build. The algorithm terminated if the desired precision $\epsilon =10^{-2}$ was reached, i.e., the difference between lower and upper bound of the ERT was smaller than $\epsilon$. In the sixth column, we give these lower and upper bounds of the minimal ERT. The precise optimal ERT is guaranteed to lie in this interval. The next column gives the optimal regions for *p* which guarantee the former ERT. The last column gives the total time (in seconds) needed to compute the results. This time comprises building the PMC, extracting the bisimulation quotient and iteratively computing the optimal parameter regions. The second row for each number of processes (depicted in italics) gives results for the *worst-case* recovery time ($\text {WC}_\text {opt}$) instead of the expected recovery time.

We can see that the size of the resulting PMC is exponential in the number of processes, i.e., $2^n$ states for *n* processes. However, the PMC size after bisimulation clearly shows that a huge part of the original states are bisimilar due to the inherent symmetries in Herman’s protocol. Thus, applying bisimulation minimization is a crucial preprocessing step which is necessary to handle large rings. However, the number of transitions after bisimulation is still large compared to the number of states and the resulting PMC has a nearly complete graph leading to a dense matrix representation. The huge number of transitions makes analyzing the larger PMCs very challenging and thus, these PMCs are at the boundary of what is currently possible in probabilistic model checking [17]. As the number of transitions grows exponentially in the number of processes the time needed for analysis also increases exponentially. Whereas 15 processes can be analyzed in less than 2 min, 19 processes require over 2 h of computation.

*Comparison with* [25] We compare our results with the results given in [25]. The main difference is a slightly different measure in both computations: we focus on the *expected* recovery time (ERT) here whereas in [25], the *worst-case* (WC) recovery time was computed. The worst-case recovery times obtained by our approach are given in Table 1 (in italics). Comparing our results with the plots given in [25], it seems that both approaches yield the same optimal values. However, we explicitly give the intervals for the optimal values. This also allows a comparison between expected and worst-case recovery times. The first difference is that for 9 processes a fair coin is still optimal for the worst-case recovery time but a biased coin is better for the expected recovery time. For larger number of processes, a biased coin is optimal for both measures. However, the optimal bias differs between both measures. For the worst-case, the optimal bias is farther away from $p=0.5$ compared to the expected recovery time. For 15 processes for example, the optimal values for the worst-case recovery time are $p\approx 0.26$ and $p\approx 0.74$ whereas the optimal values for the expected recovery time are $p \approx 0.31$ and $p \approx 0.69$.

The approach in [25] plots the effect of different values of *p* by sampling a large number of parameter values. The approach can only return the best sample point but it is not guaranteed to be optimal. In contrast, our approach ensures that the returned intervals contain the optimal parameter values.

Kwiatkowska et al. [25] gave results for different parameter values for up to 15 processes. In comparison, we were able to push the current state-of-the-art to 19 processes. Note that [25, Fig. 3] does contain values for up to 21 processes, but only for a fair coin $p=0.5$. This is computationally simpler because of the additional symmetry $p = 1-p$. In contrast, we compute results for all possible coin biases.

**Fig. 8:**
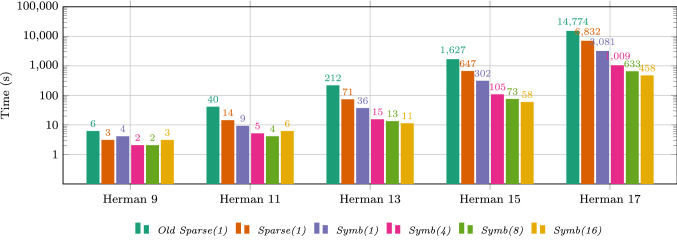
Timings for different configurations of the parameter synthesis algorithm on Herman’s protocol

### Timings of the parameter synthesis algorithm

The timings of different configurations of our algorithm for increasing number of processes are depicted in Fig. 8. We evaluate six different configurations of our parameter synthesis algorithm and display the total time in a logarithmic scale. Configuration *Old Sparse(1)* is the original algorithm used in previous work [2]. This configuration uses a sparse matrix representation of the PMC and is single-threaded. All remaining configurations use the improved version of the algorithm as presented in Sect. 4. Configuration *Sparse(1)* is most similar to the original algorithm as it also uses a sparse matrix representation with one thread. Configuration *Symb(1)* is single threaded as well but initially builds the PMC in a symbolic representation using binary decision diagrams (BDDs). During bisimulation minimization a sparse matrix representation is constructed [19]. This workflow allows to quickly build the (large) original PMC in a memory saving format and only use the memory exhaustive matrix representation for the (smaller) bisimulation quotient. The remaining configurations *Symb(4)*, *Symb(8)* and *Symb(16)* exploit the parallelization of the algorithm by using 4, 8, and 16 cores, respectively.

**Fig. 9:**
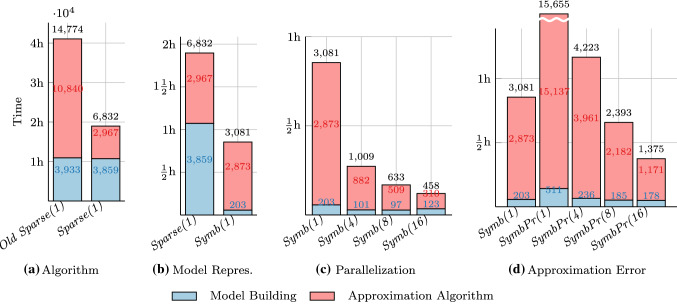
Detailed timings for different configurations of the parameter synthesis algorithm on Herman 17 (color figure online)

One can clearly see that the new algorithm significantly improves upon the original one. Comparing *Old Sparse(1)* and *Sparse(1)* which only differ in the used algorithm one can clearly see that the improved algorithm is more than two times faster. Using a symbolic representation improves the runtime further. For Herman’s protocol with at least 13 processes, *Symb(1)* is more than two times faster than *Sparse(1)*. Lastly, the parallelization again decreases the runtime. If we compare for example *Symb(4)* to the original *Old Sparse(1)* on Herman 13 and larger, the three main changes—improved algorithm, symbolic building and parallelization—yield a more than 14 times faster analysis. Instead of over 4 h for Herman 17, the result can now be obtained within 17 min.

We provide a detailed breakdown of the timings on Herman’s protocol with 17 processes in Fig. 9 and compare the different configurations. For each configuration we show the total time (in seconds) on top of the corresponding bar. Additionally, we distinguish two separate timings:

1. *Model building* (in blue): the time needed to build the PMC (either in symbolic or sparse representation) and applying the bisimulation minimization,
2. *Approximation algorithm* (in red): the time needed to perform the parameter synthesis via the approximation algorithm.

Note that the total time also contains timings for e.g., distributing the PMC to all cores for parallel computation and therefore is slightly larger than the sum of both build and analysis time.

Figure 9a compares the old algorithm *Old Sparse(1)* from [2] to the improved *Sparse(1)*. We can again clearly see how the revised algorithm shortens the analysis time by a factor 3.7 while the building time stays the same.

Figure 9b compares both model representations. Configuration *Sparse(1)* uses a sparse matrix representation while *Symb(1)* initially starts with a symbolic BDD representation. Comparing both configurations clearly shows the large influence of using the right model representation. Switching to a symbolic representation yields a 20 times faster model building. Note that for *Symb(1)*, building the initial PMC requires only 3s and the remaining 200s are spent on the bisimulation minimization.

Figure 9c compares the effect of parallelizing the algorithm. We compare *Symb(1)* with the parallelized variants *Symb(4)*, *Symb(8)* and *Symb(16)* using 4, 8, and 16 cores, respectively. Parallelization yields performance speed-ups nearly linear in the number of cores. For example, the approximation algorithm runs 3.25 times faster on 4 cores. Moreover, the underlying operations for constructing and reasoning about BDDs are also performed on multiple cores, thanks to functionality included in the multi-core BDD library Sylvan [30]. As a result, using 4 cores instead of 1 core for model building also reduces the building time by 50%. Using more than 4 cores does not seem to improve the building time any further, most likely because the algorithm for bisimulation minimization is still sequential.

**Fig. 10:**
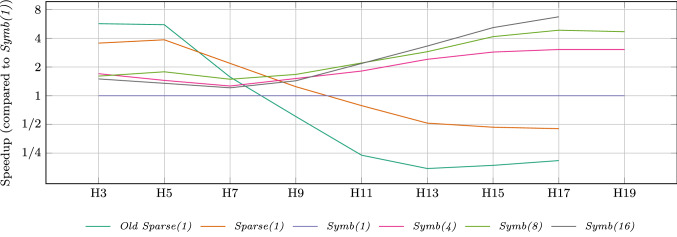
Speed-up for for different variants of the algorithm on Herman’s protocol

**Fig. 11:**
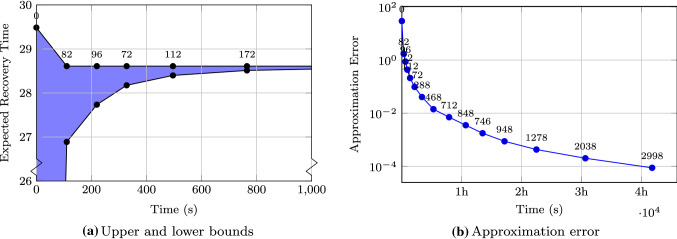
Detailed timings for the iterations in one run of the algorithm on Herman 17

Finally, Fig. 9d shows the influence of increasing the desired precision from $\epsilon =10^{-2}$ to $\epsilon =10^{-4}$. Here, configuration *Symb(1)* is the baseline as before, whereas the other four configurations *SymbPr(1)*, *SymbPr(4)*, *SymbPr(8)* and *SymbPr(16)* are the more precise configurations which only terminate if the approximation error is less than $10^{-4}$. We see that increasing the required precision $\epsilon$ by a factor 100 leads to an increase in analysis time by a factor 4. However, using more cores can mitigate this effect. The parallelization becomes nearly perfectly linear for the higher precision: doubling the number of cores reduces the analysis time by 50%. This shows that the parallelization of our algorithm comes with nearly no overhead and the cores are never idle.

Figure 10 shows the speed-up of all six considered configurations. The scale is logarithmic and configuration *Symb(1)* is used as baseline. We discuss the trends for larger numbers of processes ($N>9$). We can again see how using a sparse (instead of symbolic) representation leads to a decreased performance. Combining the sparse PMC with the old algorithm reduces the performance to one fourth of the baseline. On the other side, using parallelization increases the speed-up compared to a single process. Using 4 cores leads to a speed-up of up to factor 3, 8 cores yield a speed-up of up to 4.5 and 16 cores yield a speed-up of up to 6. Note that these speed-ups are not optimal. While with more cores the analysis times gain a nearly perfect speed-up, the model building can not be parallelized as efficiently.

Lastly, Fig. 11 considers the number of iterations of the approximation algorithm and the correspondence to the approximation error for configuration *SymbPr(1)* on Herman 17. Figure 11a plots the current upper and lower bound of the ERT over the execution time of the approximation algorithm. Each dot represents one iteration and is labeled with the number of PLA calls within this iteration. In the beginning (iteration 0), we choose zero as lower bound and obtain an upper bound by sampling. This first upper bound is already very close to the actual ERT and differs by only 3%. In general, the initial sampling considers between a dozen and a hundred sample points—depending on the number of initial regions—and in most cases yields results close to the actual ERT. After the first iteration, we also obtained a first lower bound and the upper bound is already very near to the optimal ERT. We can see that in further iterations mostly the lower bound is improved while the upper bound remains nearly unchanged. Thus, using the upper bound obtained after a couple of iterations should already be very close to the optimal ERT. The remaining time is spent improving the lower bound by refining the parameter regions.

Figure 11b plots the difference between upper and lower bounds over the execution time of the algorithm. Each dot again represents one iteration and gives the number of PLA calls per iteration. As in the previous figure, we can see that the approximation error decreases very fast in the beginning. After 13 min the precision is already smaller than 0.1 and after 47 min it is smaller than $10^{-2}$. However, reaching a precision of $10^{-4}$ takes more than 4 h. Later iterations also take longer as far more regions must be checked. While for example, the first iterations need to check fewer than 100 regions, the last iterations check 20 times as many regions. In general, reaching a good approximation with an error of less than 0.1 can be reached within minutes while obtaining more precise bounds might take several hours.

### Speed reducer for Herman’s algorithm

Previously, we considered Herman’s algorithm in the *random bit* interpretation (cf. Algorithm 1) and showed that using different biases yields optimal expected recovery time for different sizes of networks. The question however remains, whether we can further improve the ERT for Herman’s algorithm. We, therefore, consider several variants of the algorithm in the following.

**Fig. 12:**
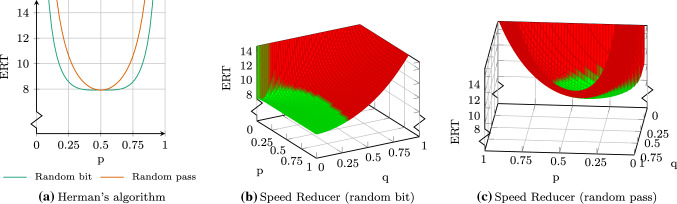
Expected recovery times for different variants of Herman’s protocol for 9 processes (color figure online)

The first variant is a slightly different formalization of the algorithm and is given in Algorithm 4. Here, with probability *p* the token is passed along by flipping the value $x_i$ of the current process. With probability $1-p$ the token is kept and $x_i$ remains unchanged. We call this variant the *random pass* interpretation of Herman’s algorithm [25]. It was already stated in [25] that for a fair coin with $p=0.5$ both interpretations *random bit* and *random pass* coincide. However, for biased coins this is not the case. This can be seen in Fig. 12a, where we plotted the ERT of both interpretations for Herman 9. For $p=0.5$ both interpretations yield the same result, but for other values of *p*, the random bit interpretation performs better. Moreover, the random bit variant yields optimal results for biased coins with $p=0.458$ and $p=0.542$ which are overall better then the results for random pass.
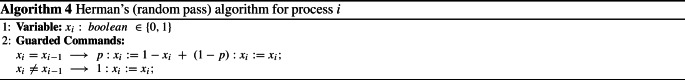

Another possible improvement is to place a *speed reducer* [13] on top of Herman’s algorithm (cf. Algorithm 2). We extend both interpretations of Herman’s algorithm with a speed reducer. Figure 12b shows the ERT for a speed reducer on top of Herman’s random bit interpretation. The area colored in green indicates ERT values which are better than the optimal ERT of the random bit variant without speed reducer. The best values can be achieved if *q*—the probability to switch to speed-reducer mode—is small. The value of *p*—the probability to switch back from speed-reducer mode—is not as relevant but should also be small. Using a speed reducer, we, thus, can further improve the ERT but require 1 bit of additional memory, i.e., double the memory consumption.

Figure 12c shows the speed reducer on top of the random pass interpretation. Again, using a speed reducer improves the ERT. In contrast to the previous variant, the random pass speed reducer obtains optimal values for values of $q \approx 0.25$ and $p \approx 0.1$.

We also created a modified variant of the speed reducer, called *SR2*. The steps of *SR2* are given in Algorithm 5. This variant is based on the regular speed reducer (cf. Algorithm 2), but has a different behavior in speed-reducer mode. Whereas normally the token is not passed along in speed-reducer mode, in *SR2* it is passed along with probability *r* and kept with probability $1-r$. *SR2*, therefore, combines all features of the regular protocol—the probability *r* to pass along a token—and the speed reducer—the two different modes. The protocol requires three parameters which can be optimized.
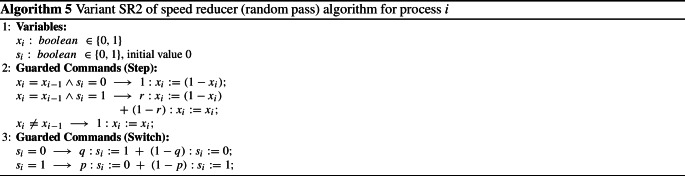

**Table 2:** Results for different variants of Herman’s protocol (including speed reducer)

Model		PMC		PMC (bisimulation)		Results for Symb. P4 (1E−2)		
Variant	Size	# States	# Trans.	# States	# Trans.	${\mathrm{ERT}_\mathrm{opt}}$	Opt. parameters	Time
Random bit	7	129	2316	16	137	[4.485, 4.493]	$p=0.498$	1 s
Random pass		129	2316	11	73	[4.493, 4.493]	$p=0.500$	1 s
Bit SR		312,705	2,455,342	19,867	155,384	[4.330, 4.337]	$p=0.087$, $q=0.060$	83 s
Pass SR		129,697	816,640	4243	31,619	[4.682, 4.691]	$p=0.282$, $q=0.237$	29 s
Bit SR2		312,705	2,455,342	39,722	310,766	[4.331, 4.337]	$p=0.088$, $q=0.061$, $r=0.501$	4895 s
Pass SR2		312,705	2,455,342	19,867	155,384	[4.685, 4.691]	$p=0.281$, $q=0.237$, $r=0.001$	7306 s
Random bit	9	513	20,196	55	1203	[7.914, 7.921]	$p=0.541$	2 s
Random pass		513	20,196	31	497	[7.922, 7.922]	$p=0.500$	2 s
Bit SR		10,601,985	146,249,062	568,249	7,826,811	[7.457, 7.465]	$p=0.085$, $q=0.064$	17,566 s
Pass SR		3,283,521	35,497,472	91,611	1,306,247	[6.907, 6.912]	$p=0.240$, $q=0.162$	1879 s
Bit SR2		10,601,985	146,249,062	1,136,484	15,653,845	[0, 8.205]	$p=0.500$, $q=0.250$, $r=0.500$	> 12 h
Pass SR2		10,601,985	146,249,062	568,249	7,826,811	[0, 7.498]	$p=0.250$, $q=0.250$, $r=0.250$	> 12 h
Random bit	11	2049	179,196	182	10,081	[12.097, 12.102]	$p=0.366$	5 s
Random pass		2049	179,196	95	3853	[12.206, 12.206]	$p=0.500$	5 s
Bit SR		371,185,665	9,001,561,822	–	–	–	–	> 12 h
Pass SR		86,036,097	1,590,272,000	–	–	–	–	> 12 h
Random bit	13	8193	1,602,516	625	85,293	[16.942, 16.949]	$p=0.331$	15 s
Random pass		8193	1,602,516	317	32,331	[17.346, 17.346]	$p=0.500$	23 s

We give a detailed comparison of all investigated variants for Herman’s protocol in Table 2. The first column states the considered variant of Herman’s protocol. We consider Herman’s protocol in both the random *bit* and the random *pass* variant. Both variants are also considered with a speed reducer (*SR*) on top and with the modified variant of the speed reducer (*SR2*). The second column in Table 2 indicates the number of processes. The next four columns indicate the number of states and transitions of the PMC after building and after applying bisimulation minimization, respectively. The last three columns give the results for applying the parameter synthesis algorithm. We used the configuration with symbolic model building, 4 parallel processes and $\epsilon =10^{-2}$. The three columns give the optimal ERT, the optimal parameter values and the time (in seconds) required to obtain these results. Note that multiple regions can contain optimal parameter values. For the sake of simplicity, we, therefore, output the best sample value which guarantees the upper bound.

First of all, we see that using a speed reducer increases the size of the resulting PMC by several orders of magnitude. This is due to the additional memory of 1 bit to keep track of the current mode and to the change between the speed-reducer modes which requires additional transitions and therefore also additional states. Basically, we get a copy of each PMC state for each speed-reducer mode. For 11 processes, the PMCs contain more than 1 billion transitions and cannot be analyzed further.

The results indicate that the *random bit* variant gives better results than the *random pass* variant. For increasing number of processes this difference becomes larger. The random bit interpretations also profits from a biased coin whereas the random pass variant yields the optimal results for a fair coin with $p=0.5$.

**Fig. 13:**
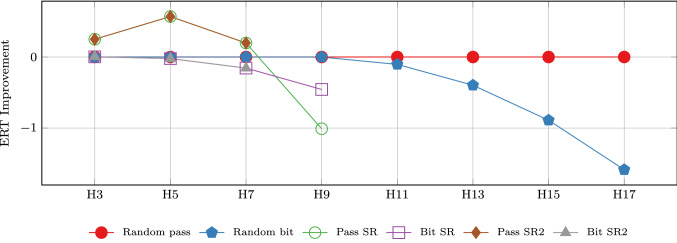
Improvements on ERT (compared to fair coin) for different variants of the algorithm on Herman’s protocol

The speed reducer variants significantly improve the ERT compared to the original protocol. For example, for 9 processes the *random pass speed reducer* variant is 12% better than the original variant. Interestingly, for 9 processes the *random pass speed reducer* variant is better than random bit SR—whereas in the original protocol it is the other way round. The variants *SR2* with one additional parameter do not further improve the ERT and yield exactly the same results as the *SR* variant. Thus, adding more randomization to the protocol does not change the ERT any more.

The timings for the analysis of the speed reducer variants are orders of magnitude larger than for the original variants. This is mostly due to the increased state space sizes. Additionally, the *SR2* variants need again orders of magnitude more time than the *SR* variants. While the state spaces sizes are similar between *SR* and *SR2* the additional overhead comes from the additional third dimension in the parameter space. The additional parameter yields 8 new regions after every split—compared to only 4 regions in 2 dimensions.

The last plot in Fig. 13 visualizes the improvements for the ERT for all considered variants of Herman’s protocol. We use the *random pass* interpretation with a fair coin as baseline and depict the (absolute) differences for increasing number of processes. As stated before, the *random bit* interpretation with biased coin clearly improves the ERT and yields significantly better results for large number of processes. The speed reducer variants are even better and the *random pass SR* yields the overall best results. The *SR2* variants yield the same results as *SR* and are therefore indistinguishable.

**Table 3:** Results for different variants of Dijkstra’s algorithm (including speed reducer)

Model		PMC		PMC (bisimulation)		Results for Symb. P4 (1E−2)		
Variant	Size	# States	# Trans.	# States	# Trans.	${\mathrm{ERT}_\mathrm{opt}}$	Opt. parameters	Time
Dijkstra3	3	28	57	3	4	0.111		1 s
Dijkstra3 SR		736	1647	5	6	0.111		18 s
DijkstraK		28	72	4	7	0.481		1 s
DijkstraK SR		649	1485	17	28	[0.481, 0.487]	$p=0.875$, $q=0.125$	1 s
Dijkstra3	5	244	783	52	181	2.500		1 s
Dijkstra3 SR		65,023	281,352	10,517	44,722	[2.500, 2.506]	$p=0.997$, $q=0.003$	13 s
DijkstraK		3126	13,750	101	460	3.183		1 s
DijkstraK SR		602,086	2,668,345	18,318	79,792	[3.183, 3.190]	$p=0.997$, $q=0.003$	26 s
Dijkstra3	7	2188	9477	630	2839	5.574		1 s
Dijkstra3 SR		5,071,342	39,399,081	1,265,270	9,750,534	[5.574, 5.580]	$p=0.999$, $q=0.001$	10,801 s
DijkstraK		823,544	5,176,556	3260	20,899	7.357		54 s
DijkstraK SR		1,286,682,888	10,165,883,385	–	–	–	–	MO
Dijkstra3	9	19,684	107,163	6194	34,278	9.027		4 s
Dijkstra3 SR		399,172,324	5,476,666,185	–	–	–	–	MO
DijkstraK		387,420,490	3,185,457,354	–	–	–		MO
DijkstraK SR		5,167,625,026,906	71,611,028,231,841	–	–	–	–	MO

*In conclusion, using a speed reducer seems promising to improve the expected recovery times in Herman’s protocol.*

### Speed reducer for Dijkstra’s algorithm

As an additional example, we analysed Dijkstra’s self-stabilizing algorithm [11]. We consider two variants: the algorithm with a 3-state machine and the one with a *K*-state machine where $K=N$ equals the number of processes *N*. Note that both variants are deterministic algorithms and therefore require a dedicated process which behaves differently than the other processes. We also consider variants where we extended both algorithms with a speed reducer.

The results are given in Table 3. We see that using a speed reducer does not improve the ERT of Dijkstra’s algorithm. The optimal parameter values are such that the speed-reducer mode is nearly never entered ($q \approx 0$) and also immediately left again ($p \approx 1$).

We can only analyze the variants for a small number of processes ($N \le 5$), because the state space sizes of the resulting PMCs explode. For the larger PMCs we run into a memory out (MO). Additional state space reduction techniques such as symmetry reduction are required to support the analysis for larger number of processes. Note that for all considered protocols, the bottleneck of the analysis is not our synthesis algorithm, but the building of the model. In general, if we obtain a simplified PMC, our algorithm is able to compute the optimal parameter regions.

## Related work

### Model repair in probabilistic systems

In [5], the authors modify the probability of controllable transitions to achieve a new model of the program that satisfies a desired property represented in the form of a rational function over a set of parameters while minimizing the cost function. They use the state elimination method presented in [8] to obtain the rational parametric function. They show that this problem can be reduced to a non-linear optimization problem. Their work is similar to the second approach from our previous paper [2] which also uses the rational function. However as seen in [2] and proven in [22], this approach does not scale well making the use of approximation methods necessary. While they present a solution to the model repair problem, their work differs from ours in two aspects. First, they consider only one initial state. This significantly reduces the memory and computation time. Second, their solution works for models representing a single process or networks that are not necessarily anonymous since their approach does not guarantee the preservation of anonymity of processes. Recall that the TPM of a distributed program is a function of the TPMs of the underlying processes which is not accounted for in this work.

In [27], instead of performing non-linear optimization as done in [5], which is not scalable, the authors take a greedy approach to finding the optimal evaluation of parameters that results in satisfying the property. This greedy approach is correct under monotonicity of the parameters. Our work is different from this work as we are not only interested in satisfying the property, e.g., having a recovery time below a certain threshold, but in achieving the optimal recovery time. Moreover, we approximate the optimal numerical values for parameters up to a desired precision and do not rely on monotonicity. Also, in [7], the authors study an orthogonal problem, where they use abstraction-refinement in order to tackle the state explosion problem in model repair. We, however, use parameter regions in order to find optimal bias. One can of course combine the two techniques to gain more scalability but this is outside the scope of this paper.

In [21], the authors analyze the fault-tolerance of inter-process communication in a space probe. They use parametric probabilistic model checking to compute rational functions for the availability of the space probe and find the optimal parameter values. Their approach is similar to the second approach from [2]. Due to the scalability issues with the approach the authors restrict their analysis to one parameter. In contrast, our approximation approach also works for multiple parameters and allows to analyze larger models with significantly more states.

### Analysis of self-stabilizing algorithms

In [25], the authors verified the asymptotic bounds on the worst-case recovery time of Herman’s token circulation algorithm with probabilistic model checking. By calculating the worst-case expected recovery time for different probabilities and network sizes, they made an interesting and surprising observation that a fair coin does not lead to minimum worst-case expected recovery time for networks of size greater than 9. In this paper, for each network size, we focus on the average-case expected recovery time of the algorithm instead of the worst-case. Moreover, the analysis in [25] was performed by sampling and therefore cannot guarantee finding the optimal parameter value. In contrast, our approach computes bounds for the exact optimum which can be refined up to the desired precision.

In [31], the authors approached our problem with genetic algorithms. Although their results may be obtained using our approach as well, the drawback of using genetic algorithms is that there are no guaranteed theoretical bounds on the optimality of the result. In contrast our approximation approach gives sound error bounds on the result.

Finally, in [15], the authors studied the role of different parameters on the convergence time of self-stabilizing systems using probabilistic model checking. In particular, they showed that the asymptotic worst-case complexity is not necessarily the best metric to characterize the performance of self-stabilizing systems. The parameters studied include the type of faults, place of occurrence of faults, etc. Also, in [14], the authors used the same technique to study the performance of weak-stabilizing algorithms. In [1], the effect of schedulers, not the internal behavior of the program, on the possibility and speed of convergence is studied through an empirical study.

## Conclusion

In this paper, we proposed an automated method to compute the probability values that result in the minimum average recovery time in a given randomized self-stabilizing distributed algorithm. We call this the *optimal bias synthesis problem*. This work is based upon [2]. While [2] presented three different solution approaches to the parameter synthesis problem, in this work we focus on the most promising approach based on parameter lifting [29] and significantly improve it compared to the original approach. Our algorithm works as follows. First, we transform a given randomized self-stabilizing algorithm into a parametric Markov chain (PMC). Next, to compute the best probabilities, we compute over- and under-approximations of the average recovery time for all parameter values. By iteratively refining parameter regions which lead to small convergence times, the optimal probabilities can be approximated up to the desired precision. Our algorithm exploits multi-core platforms by evaluating independent regions in parallel.

Compared to [2], we delve deeper into evaluating the performance of randomized self-stabilizing algorithms. First, our results systematically confirmed the previous empirical method [25] that a fair coin ($p=0.5$) does not necessarily yield minimum expected recovery time in Herman’s randomized self-stabilizing token circulation. Given the observed trend in our experiments, we conjecture that as the network size grows, increasing the bias becomes more effective. We also showed that our parallelization yields speed-ups nearly linear in the number of cores. Furthermore, we compared different techniques of model building and showed that a combination of BDD-based model building with sparse matrix computations significantly outperform explicit-state techniques. Finally, we studied the impact of composing speed reducers [13] with Herman’s algorithm and identified probabilities that improve the performance of Herman’s algorithm.

Future work includes the study of the problem in the context of other distributed algorithms such as randomized leader election and consensus, and for probabilistic programs [16]. One can also study the same problem in the presence of different scheduling schemes (modeled as a Markov decision process). A more challenging avenue of research is to not only parameterize the probability function, but also make the computational model parametric in terms of the number of processes. Finally, we can use our techniques to automatically generate state encoding [14, 15] schemes to orthogonally improve the recovery time.

## Footnotes

1 https://www.prismmodelchecker.org/doc/semantics.pdf.

2 A probabilistic scheduler chooses actions in a fully probabilistic way (i.e., there is no nondeterministic choice of distributions as in MDPs). Hence, systems that work under a fully probabilistic scheduler can be modeled by DTMCs regardless of whether they are synchronous or asynchronous.

3 https://github.com/moves-rwth/optimal-bias-synthesis.

4 https://moves-rwth.github.io/stormpy/.
